# Associations between vascular health, brain stiffness and global cognitive function

**DOI:** 10.1093/braincomms/fcae073

**Published:** 2024-02-29

**Authors:** KowsalyaDevi Pavuluri, John Huston, Richard L Ehman, Armando Manduca, Clifford R Jack, Matthew L Senjem, Prashanthi Vemuri, Matthew C Murphy

**Affiliations:** Department of Radiology, Mayo Clinic, Rochester, MN 55905, USA; Department of Radiology, Mayo Clinic, Rochester, MN 55905, USA; Department of Radiology, Mayo Clinic, Rochester, MN 55905, USA; Department of Radiology, Mayo Clinic, Rochester, MN 55905, USA; Department of Physiology and Biomedical Engineering, Mayo Clinic College of Medicine, Rochester, MN 55905, USA; Department of Radiology, Mayo Clinic, Rochester, MN 55905, USA; Department of Information Technology, Mayo Clinic, Rochester, MN 55905, USA; Department of Radiology, Mayo Clinic, Rochester, MN 55905, USA; Department of Radiology, Mayo Clinic, Rochester, MN 55905, USA

**Keywords:** vascular brain injury, cognitive impairment, magnetic resonance elastography, brain stiffness, dementia

## Abstract

Vascular brain injury results in loss of structural and functional connectivity and leads to cognitive impairment. Its various manifestations, including microinfarcts, microhaemorrhages and white matter hyperintensities, result in microstructural tissue integrity loss and secondary neurodegeneration. Among these, tissue microstructural alteration is a relatively early event compared with atrophy along the aging and neurodegeneration continuum. Understanding its association with cognition may provide the opportunity to further elucidate the relationship between vascular health and clinical outcomes. Magnetic resonance elastography offers a non-invasive approach to evaluate tissue mechanical properties, providing a window into the microstructural integrity of the brain. This retrospective study evaluated brain stiffness as a potential biomarker for vascular brain injury and its role in mediating the impact of vascular dysfunction on cognitive impairment. Seventy-five participants from the Mayo Clinic Study of Aging underwent brain imaging using a 3T MR imager with a spin-echo echo-planar imaging sequence for magnetic resonance elastography and T_1_- and T_2_-weighted pulse sequences. This study evaluated the effects of vascular biomarkers (white matter hyperintensities and cardiometabolic condition score) on brain stiffness using voxelwise analysis. Partial correlation analysis explored associations between brain stiffness, white matter hyperintensities, cardiometabolic condition and global cognition. Mediation analysis determined the role of stiffness in mediating the relationship between vascular biomarkers and cognitive performance. Statistical significance was set at P-values < 0.05. Diagnostic accuracy of magnetic resonance elastography stiffness for white matter hyperintensities and cardiometabolic condition was evaluated using receiver operator characteristic curves. Voxelwise linear regression analysis indicated white matter hyperintensities negatively correlate with brain stiffness, specifically in periventricular regions with high white matter hyperintensity levels. A negative association between cardiovascular risk factors and stiffness was also observed across the brain. No significant patterns of stiffness changes were associated with amyloid load. Global stiffness (µ) negatively correlated with both white matter hyperintensities and cardiometabolic condition when all other covariables including amyloid load were controlled. The positive correlation between white matter hyperintensities and cardiometabolic condition weakened and became statistically insignificant when controlling for other covariables. Brain stiffness and global cognition were positively correlated, maintaining statistical significance after adjusting for all covariables. These findings suggest mechanical alterations are associated with cognitive dysfunction and vascular brain injury. Brain stiffness significantly mediated the indirect effects of white matter hyperintensities and cardiometabolic condition on global cognition. Local cerebrovascular diseases (assessed by white matter hyperintensities) and systemic vascular risk factors (assessed by cardiometabolic condition) impact brain stiffness with spatially and statistically distinct effects. Global brain stiffness is a significant mediator between vascular disease measures and cognitive function, highlighting the value of magnetic resonance elastography-based mechanical assessments in understanding this relationship.

## Teaser

Pavuluri *et al*. investigated brain stiffness as a potential new biomarker for vascular brain injury, using magnetic resonance elastography. They found brain stiffness relates to vascular health and cognitive function, mediating the impact of white matter hyperintensities and cardiovascular conditions on cognition, suggesting its diagnostic potential for vascular cognitive impairments.

## Graphical Abstract

**Graphical Abstract:**
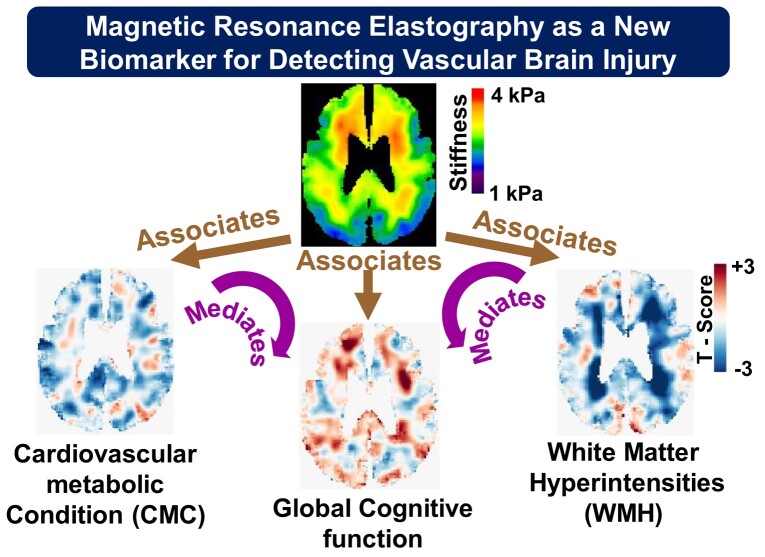

## Introduction

Vascular brain injury encompasses a wide range of disease processes related to structural alterations and dysfunction of the cerebral vasculature, ultimately impacting the cognitive function. These processes include clinically asymptomatic cerebral infarctions, white matter (WM) hyperintensities (WMH), microinfarctions and microbleeds, as well as more general changes in the brain such as blood–brain barrier dysfunction, impaired interstitial fluid drainage, altered cerebral blood flow and microstructural myelin injury.^1^ In epidemiological clinical–pathological studies of aging, cerebrovascular pathologies such as gross infarcts and moderate-to-severe atherosclerosis, arteriolosclerosis and cerebral amyloid angiopathy have been observed in 20–30% of brains examined.^2,3^ These pathologies are found to be independently associated with cognitive decline in late life.^2^ Irrespective of the underlying mechanism, the terms ‘vascular brain injury’ and ‘vascular cognitive impairment’ are commonly used to describe cerebrovascular diseases.^4,5,6-10^ Neurovascular dysfunction has been increasingly recognized to impact cognitive outcomes when coexistent with Alzheimer’s disease (AD) pathologies .^11,12^ Several studies have shown that vascular abnormality may be an early event in AD that promotes the cognitive decline and lowers the threshold of Aβ burden to cause clinically overt dementia.^13,14^ The heterogeneity of cerebrovascular disease and coexistence of proteinopathy, especially with AD, makes it challenging to elucidate the neuropathological substrates and mechanisms of vascular degeneration.^14-16^ Recent advances in neuroimaging, neuropathology, epidemiology and genetics have expedited the understanding of the relationship between vascular injury and cognitive function.

WMH, microbleeds, microinfarcts, cortical superficial siderosis, widened perivascular spaces and large infarcts are commonly used imaging markers to identify vascular brain injury.^1^ Of these, WMH are the most widely utilized^17,18^ markers of vascular changes in the brain, although these changes are reported to occur even before WMH become detectable.^19^ WMH may represent only the extreme end of a continuous spectrum of WM injury, creating a need for imaging approaches that can detect more subtle changes.^17^ Advanced and conventional MRI methods have been investigated for this application including diffusion tensor imaging (DTI), arterial spin labelling, T_2_-weighted MRI and volumetric measurements and provide quantitative measures of WM microstructural integrity, cerebral blood flow and dilated perivascular spaces.^1^ Effective early diagnosis of vascular dysfunction is necessary in order to implement lifestyle modifications that delay the onset and the progression of various age-related dementia and stroke.^20^ Moreover, the relationship between current measures of vascular health and clinical outcomes remains probabilistic, reflecting an incomplete understanding of the mechanisms that link the two and suggesting that additional biomarkers may be beneficial in bridging the gap.

Over the last decade, magnetic resonance elastography (MRE) has proved to be a reliable non-invasive imaging technique to measure brain viscoelastic properties. Viscoelastic properties provide insights into microstructural integrity of brain tissue and reflect the rigidity and organization of neurons, axons and extracellular matrix (ECM).^21-23^ MRE has been applied to assess the viscoelastic changes in aging brain and several neurodegenerative conditions including Alzheimer’s disease, frontotemporal dementia, multiple sclerosis and dementia with Lewy bodies.^24-31^ Most of these studies reported mechanical property differences in the whole brain and hippocampal, lobar and cortical grey matter regions.^32-34^ Recent advances in high-resolution MRE techniques have demonstrated that mechanical biomarkers are related to cognitive function,^35-37^ motivating further study on the use of MRE as a clinical biomarker in assessing aging and neurodegeneration. Viscoelastic properties, specifically damping ratio of hippocampal subfields, were shown to associate with aspects of memory in young adults.^29,34^ In later studies by the same group, brain stiffness and cognition were shown to relate in paediatric cerebral palsy and adolescent risk-taking tasks.^38,39^ Furthermore, a detailed assessment of the relation between hippocampal subfield stiffness and specific aspects of memory was reported.^37^ However, less is known about the relationship between mechanical properties and cognitive function in older adults and how this relationship is related to specific pathologies that can be measured by other modalities.

Therefore, the hypothesis of this study is that MRE-based biomarkers can provide a global indication of brain vascular health. To this end, we evaluated the association between brain stiffness with cerebrovascular disease, systemic vascular health, amyloid load and cognition. In addition, we assessed the mediatory role of stiffness as a marker of vascular disease and its impact on cognition.

## Methods and materials

### Study participants

After obtaining approval from the Mayo Clinic Institute Review Board and written informed consent, data were acquired in 75 participants including fluid-attenuated inversion recovery (FLAIR) MRI, MRE examinations, PET, vascular health clinical record summary and cognitive assessments. Participants were enrolled from the Mayo Clinic Study of Aging (MCSA) and Alzheimer’s Disease Research Center, including 44 cognitively unimpaired participants free of significant amyloid load and 31 along the Alzheimer’s disease spectrum with significant amyloid burden. MRE data of all the participants were acquired as a part of previous aging and AD studies.^26,32^

## Data acquisition

### Anatomic MRI

For assessing cerebrovascular disease, MRI data were obtained for each participant on a 3T GE MRI scanner using T_1_-weighted magnetization-prepared rapid gradient echo (MPRAGE) and FLAIR sequences to segment the WMH.^40^ Three-dimensional T_1_-weighted images were acquired with 1.05 mm in-plane resolution and 1.2 mm interslice spacing. Imaging parameters were repetition time (TR)/echo time (TE), 7.0/2.8 ms; 11° flip angle; inversion time, 400 ms; field of view (FOV), 27 cm; imaging matrix, 256 × 256; bandwidth, ±31.25 kHz; 1.75× array spatial sensitivity encoding technique acceleration; and 200 slice locations in sagittal orientation and superior–inferior frequency-encoding direction. Acquisition parameters for FLAIR MRI scans were TR = 11 s, TE = 147 ms, inversion time = 2250 ms, 256 × 192 matrix, 24 cm FOV and 3 mm slice thickness.

### MRE imaging

MRE acquisitions were performed on the same 3T MR scanner (SIGNA Excite, GE Healthcare, Waukesha, WI) with an 8-channel receive-only head coil using a modified flow-compensated, spin-echo echo-planar imaging pulse sequence.^41,42^ Displacement images with 3 mm isotropic resolution were collected at 60 Hz vibration frequency for eight evenly spaced phase offsets in <7 min for each participant. MRE imaging parameters including TR = 3.6 s; TE = 62 ms; FOV = 24 cm; BW = ±250 kHz; 72 × 72 imaging matrix reconstructed to 80 × 80; frequency encoding in the right-to-left direction; 3× parallel imaging acceleration; 48 contiguous 3-mm-thick axial slices; ±*x*, ±*y* and ±*z* motion encoding gradients with amplitude of 4 G/cm; and duration of 18.2 ms were used on each side of the refocusing radio frequency pulse.

### Amyloid PET imaging

Amyloid levels were assessed using PET imaging with Pittsburgh compound B (PiB), as described in detail previously.^43^ A global PET standardized uptake value ratio (SUVr) was calculated by averaging the median uptake in prefrontal, orbitofrontal, parietal, temporal, anterior and posterior cingulate and precuneus regions of interest (ROIs) weighted by voxel number. This value was then normalized to the cerebellar crus grey median using an in-house pipeline built with Statistical Parametric Mapping (SPM12). To meet the model assumptions, amyloid deposition was initially log-transformed and subsequently standardized, such that a unit increase corresponds to 1 standard deviation (SD).

### Cardiometabolic condition score

Systemic cardiovascular health was assessed from the clinical record as the summation of seven cardiovascular and metabolic conditions proposed by the US Department of Health and Human Services in 2010: hypertension, hyperlipidaemia, cardiac arrhythmias, coronary artery disease, congestive heart failure, diabetes mellitus and stroke.^44-47^

### Cognitive performance tests

All participants underwent a neuropsychological battery consisting of nine tests covering four cognitive domains: executive (Trail Making Test Part B and Wechsler Adult Intelligence Scale—Revised Digit Symbol), language (Boston Naming Test and category fluency), memory [Wechsler Memory Scale—Revised Logical Memory II (delayed recall), Wechsler Memory Scale—Revised Visual Reproduction II (delayed recall) and Auditory Learning Verbal Test delayed recall] and visuospatial performance (Wechsler Adult Intelligence Scale—Revised Picture Completion and Block Design).^48,49^ Individual global *Z*-score was estimated from the *z*-transformation of the four-domain average *Z*-score including memory, language, attention and visuospatial performance. Analyses involving cognitive function were performed in a subset of 66 participants who completed all tests within 1 year of the MRE exam.

### Image processing

T_2_-weighted FLAIR WMH were segmented with a previously described semiautomated in-house algorithm.^40^ In brief, the FLAIR and MPRAGE scans of all study participants were registered and aligned to a common template using a unified segmentation algorithm in SPM12.^50^ The FLAIR images were then masked using the WM mask from the MPRAGE image to remove non-brain tissue and voxels other than WMH. A semiautomated clustering technique was used to segment the WMH voxels on the FLAIR images, as described in a previous study.^40^ These custom masks were further reviewed and edited by trained analysts to remove any artefacts from the WMH volume. The final WMH measure used in this study was the log transform of the fraction of WMH volume relative to the total intracranial volume.

Stiffness maps were computed using neural network inversion (NNI),^51^ trained using data generated by a finite difference model of harmonic motion in a linear viscoelastic, inhomogeneous and isotropic material. We generated a set of 4500 displacement fields for training, each consisting of 64 × 64 × 64 cubes at 1.5 mm isotropic resolution, which were then downsampled to 3 mm resolution to match the acquired MRE resolution. Training patches of size 11 × 11 × 11 were then randomly selected from different locations within each field, and a randomly selected mask patch drawn from the in vivo brain masks was applied to each patch. Gaussian noise was then added to these data. Subsequently, the curl of the synthetic displacement patch was computed. All components of the computed curl were the inputs of the neural network, while the true stiffness value at the centre of the patch was the target. For *in vivo* data, phase unwrapping was performed on the MRE wave images using graph cuts.^52^ For use during inversion, tissue probability maps were computed in MRE space using a unified segmentation algorithm in SPM12^50^ with an in-house template.^53,54^ T_1_-weighted images of each participant were coregistered and resliced to the MRE magnitude image. During segmentation, both MRE and T_1_ spaces were used to reduce misregistration errors between the MRE data and the computed segmentations. A brain mask was generated indicating voxels with combined grey and white matter probabilities greater than that of CSF.^51,52^ A 22-region lobar atlas was utilized to divide the phase unwrapped brain into 6 distinct processing regions including cerebellum, brainstem, the union of the frontal lobes + deep grey matter and WM + parietal lobe + occipital lobe + corpus callosum (left and right hemispheres) and the union of the occipital parietal + temporal lobes + parietal lobes (left and right hemispheres). These regions were chosen to avoid inversion across the major dural folds (the cerebral falx, tentorium, that act as wave sources) and lateral sulcus (where CSF separates anatomically distinct regions and expands due to atrophy) as described previously.^42^ In each region, the region mask was applied, and we used previously described edge-aware methods of curl computation to remove longitudinal waves.^55^ Voxels with curl estimates that fell outside the mean plus or minus 3 times the SD in a 11 × 11 × 11 voxel sliding window were removed (∼4.6% voxels). The curl images were then scaled such that the maximum amplitude of the first harmonic in the entire volume was equal to 1 (to fall within the amplitude range of the NNI training data) and sent through the neural network inversion. Stiffness was estimated separately in the 6 subregions (specified above), and final maps were computed by union of these subregion estimates. For voxels present in two regions (such as the corpus callosum), the final stiffness map was assigned the mean value of the two estimates.

### Statistical analysis

Five different statistical analyses were performed using in-house MATLAB (R2023a, MathWorks, Natick, MA, USA) and Python (version 3.10, Python Software Foundation) scripts. First, voxelwise linear modelling was performed to interrogate the topography of stiffness changes associated with each measure of vascular health. To this end, following spatial normalization of each participant’s stiffness map to template space,^53^ a linear model was fit to the data at each voxel with predictors including age, sex, WMH, cardiometabolic condition (CMC) and PiB. Voxels which are present in at least half of the participants were used for the voxel analysis. *T*-score maps of WMH, CMC and PiB effects on brain stiffness were computed.

For the remaining analyses, stiffness was summarized as the mean value in the cerebrum. To assess whether partial volume effects impact this summary measure, we used a previously published simulation data set to determine the correlations between estimated stiffness with both true stiffness and brain volume.^51^ A partial correlation analysis was then performed to assess the relationship between global brain stiffness (µ), WMH and CMC in the participants. In addition, the partial correlation between stiffness and global cognition was assessed in the subset of participants with neuropsychological assessments. Pairwise correlation between the residuals resulting from the linear regression between global brain stiffness (µ), WMH, CMC and cognition was calculated without controlling other variables, partial control for age and sex and full control of all covariables including amyloid load, respectively.

After finding statistically significant partial correlations between stiffness, vascular health measures and global cognition, we sought to determine if the vascular biomarkers played a mediating role in these correlations. Specifically, we conducted mediation analysis^56,57^ to investigate whether brain stiffness captured the influence of vascular disease on cognitive outcomes. Results were considered significant if the *P*-value was <0.05.

The diagnostic performance of brain stiffness for discriminating participants with WMH and CMC was evaluated using receiver operating characteristic (ROC) curves. Area under the curve (AUC) was computed at various cut-off values for each WMH and CMC. At each cut-off, a logistic regression model was fit incorporating age, sex, stiffness, CMC and amyloid load as predictors. AUC was then computed using the estimated probabilities.

## Results

### Participant characteristics:

The demographics and characteristics of all the participants are presented in Table 1 including age at the time of MRE, brain stiffness, WMH, CMC and all the cognitive measures. Pearson correlations between the pairs of variables are provided in Supplementary Fig. 1.

**Table 1:** Characteristics table of participants with mean (SD) listed for the continuous variables and count (%) for the categorical variables

Number of participants = 75	
**Demographics**	
** **Age (y)	76.25 (8.7)
** **Males, no (%)	53.3
**Cognitive measures**	
** **MMSE	27.31 (2.79)
** **Global cognition ***Z***-score	−0.42 (1.41)
** **Memory	−0.45 (1.5)
** **Language	−0.51 (1.42)
** **Attention	−0.44 (1.46)
** **Visuospatial performance	−0.04 (1.10)
**Amyloid load**	
** **PiB-SUvr	1.61 (0.46)
**Vascular degeneration measures**	
** **CMC	2.25 (1.52)
** **WMH	0.82 (0.99)
** **Global brain stiffness (k Pa)	2.51 (0.16)

### Voxelwise analysis

**Figure 1:**
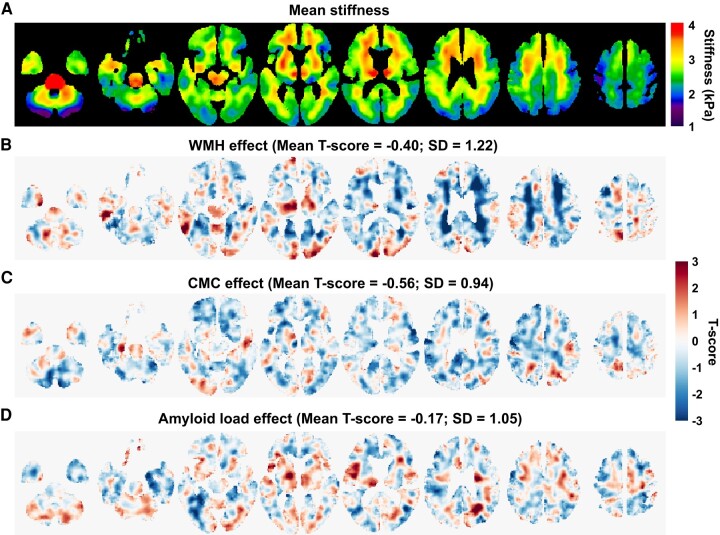
**Mean stiffness maps and voxelwise effects on stiffness.** (**A**) Mean stiffness maps at different slice locations. (**B**), (**C**) and (**D**) are *T*-score maps of estimated voxelwise effects of WMH, CMC score and amyloid load (PiB-SUVr) on stiffness, respectively. Mean and SD of *T*-score distribution over the entire brain are indicated.

Estimated mean stiffness maps from the voxelwise modelling are presented in Fig. 1A. *T*-score maps of the WMH, CMC and PiB effects on stiffness are shown in Fig. 1B–D, respectively. From the *T*-score maps of WMH, a negative association of WMH-stiffness is observed, primarily in the periventricular voxels. Mean and SD of *T*-score over the entire brain are −0.40 and 1.22. On the other hand, the CMC effect (mean *T*-score = −0.56; SD = 0.94) is associated with widespread decreased stiffness with smaller amplitude effects. No clear patterns were observed between stiffness and amyloid load. The amyloid effect on stiffness across the entire brain exhibits a relatively smaller mean *T*-score of −0.17, with an SD of 1.05. We used amyloid load as a covariable in our further statistical analysis.

### Evaluation of global stiffness estimates in simulation

**Figure 2:**
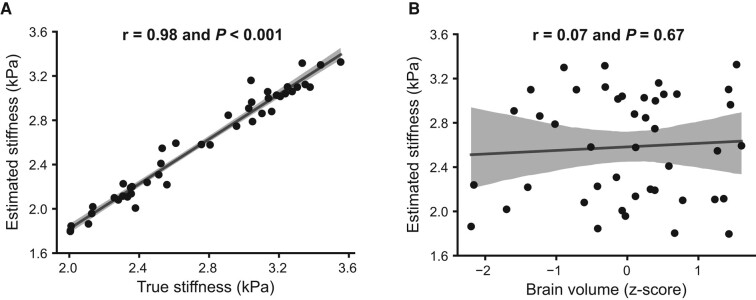
**Correlations of estimated stiffness with true stiffness and global brain volume.** (**A**) The correlation between estimated global stiffness and true stiffness. (**B**) The correlation between estimated global stiffness and global brain volume.

Correlations of estimated global stiffness with true stiffness and brain volume for the simulated data are given in Fig. 2A and B. Global stiffness estimates are highly correlated with the ground truth (*r* = 0.98, *P* < 0.001) but not significantly associated with total brain volume (*r* = 0.067, *P* = 0.67).

### Associations between brain stiffness and measures of vascular disease

**Figure 3:**
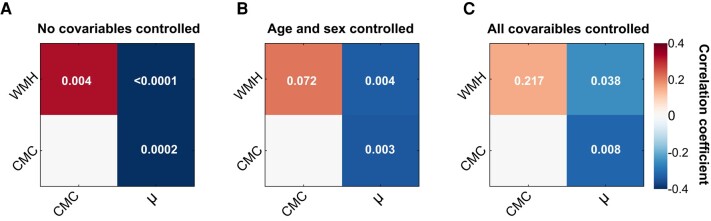
**Partial correlation analysis.** Pairwise associations between brain stiffness (µ), WMH load and CMC score. Associations are tested without controlling any covariables (**A**), controlling only age and sex (**B**) and controlling for all covariables (**C**). Statistical significance (***P***-values) of pairwise association is indicated in each box.

The results of the partial correlation analysis to assess the degree of association between the vascular biomarkers of interest are presented in Fig. 3. The degree of associations (ρ) between all possible pair of variables (µ, WMH, CMC) along with their statistical significance for three different scenarios, (i) without controlling for covariables, (ii) with control for age and sex and (iii) with control of all covariables, are shown in Fig. 3A–C, respectively. Stiffness is strongly correlated with WMH (ρ = −0.63; *P* < 0.0001), CMC (ρ = −0.41; *P* = 0.0002) and global cognition (ρ = 0.52; *P* < 0.0001) with no covariables controlled. The µ–WMH association is slightly reduced (ρ = −0.33; *P* = 0.004; ρ = −0.25; *P* = 0.038), and the statistical significance is preserved after controlling for age and sex and all covariables, respectively. When controlling for age and sex or for all covariables, we observed a slight reduction in the degree of association between stiffness and CMC (ρ = −0.34; *P* = 0.003; ρ = −0.31; *P* = 0.008, but the statistical significance is preserved. The degree of positive association between the stiffness and global cognition is reduced by half (ρ = 0.27; *P* = 0.036; ρ = 0.26; *P* = 0.046), but the statistical significance is preserved even after controlling for age, sex or all covariables. CMC and WMH are positively associated (ρ = 0.33; *P* = 0.004) without controlling for any covariables, and the statistical significance of this correlation is not preserved after controlling for the covariables.

Partial correlation between age and stiffness is much stronger (ρ = −0.6004; *P* < 10–7), indicating a more substantial association. The correlation between sex and stiffness is negligible (ρ = −0.0007; *P* = 0.9956), indicating no significant association between sex and brain stiffness in this study.

### Effect of vascular disease on cognition is explained by stiffness

The mediatory roles of stiffness, WMH and CMC in six paths with global cognition as an outcome are presented in Table 2. Each of the direct and indirect effects of the paths is assessed by controlling for age, sex, amyloid levels and the rest of the vascular biomarker covariables. Stiffness strongly mediates the effects of WMH on global cognition (*T*-score = −3.36; *P* = 0.0009) and CMC on global cognition (*T*-score = −2.10; *P* = 0.021). WMH mediates the effects of µ on global cognition (*T*-score = 2.44; *P* = 0.001). No statistically significant mediation role of WMH is observed for the relation between CMC and global cognition. There is no statistically significant mediating effect of CMC on the relationships between global cognition and µ and WMH.

**Table 2:** Mediating roles of vascular biomarkers with global cognition as outcome

Mediator	Path	*T*-score	*P*-value
**Stiffness**	WMH → global cognition	−3.36	0.0009
	CMC → global cognition	−2.10	0.021
**WMH**	µ → global cognition	2.44	0.001
	CMC → global cognition	−0.53	0.879
**CMC**	µ → global cognition	0.43	0.491
	WMH → global cognition	0.11	0.410

Both direct and indirect effects are assessed by controlling for age, sex and the rest of the covariables.

### Diagnostic accuracy of MRE stiffness for WMH and CMC

The ROC curves, as depicted in Supplementary Fig. 2, delineate the trade-off between sensitivity and specificity across a spectrum of thresholds for both WMH and CMC. For WMH, an AUC of 0.8 or above across the explored threshold range underscores a good diagnostic accuracy of MRE stiffness. On the other hand, an AUC of 0.7 or above for CMC denotes an acceptable level of diagnostic accuracy.

## Discussion

Our findings demonstrate that brain stiffness exhibits non-overlapping sensitivity to cerebrovascular disease and systemic vascular health and provides a measure of the impact of vascular health on cognitive outcomes. Voxelwise modelling illustrates that the whole brain softens with the systemic cardiovascular risk factors, and brain stiffness is negatively associated with WMH where these lesions are most often present. Partial correlation analysis between the vascular biomarkers suggests that the negative association between stiffness and vascular disease measures (both WMH and CMC) is detected with or without controlling for all the covariables (age, sex, PiB, WMH and CMC). The positive association between CMC score and WMH is statistically significant only when no covariables are controlled. Partial correlation between stiffness and WMH is statistically significant with a reduced degree of association after including age, sex, PiB and CMC. Partial correlations of stiffness with WMH and CMC are relatively moderate compared with that with age. Softening of the brain with age^26^ can confound the relationship between stiffness and WMH. Also, the association of age^58^ and vascular risk factors^13^ with WMH can weaken the correlation between stiffness and WMH. Stiffness associations with the vascular markers are significant even after controlling for amyloid load. Taken together, the findings of mediation analysis indicate that stiffness strongly mediates the effects of vascular measures on cognitive outcomes. WMH mediates the µ versus global cognition relationship, whereas CMC did not demonstrate a significant mediatory role between these biomarkers. These findings along with the ROC analysis suggest MRE brain stiffness as a promising biomarker in the evaluation of cerebrovascular disease.

The observed negative association of stiffness with vascular disease burden may indicate myelin loss, inflammation or hypoperfusion.^18,59,60^ In WMH, previous studies indicate that altered perfusion occurs due to the compromise of blood flow in cerebral vasculature and of the neurovascular unit (NVU).^61^ This alteration in perfusion is notably associated with changes in cerebral blood flow, especially in individuals with cerebrovascular risk factors.^62^ Recent arterial spin labelling (ASL) studies have shown that perivascular perfusion alterations are associated with cerebral blood flow in the aging population.^63^ Such alterations in blood flow and perfusion can have profound effects on tissue health and integrity. Hetzer *et al*.^64^ reported the significant correlation between perfusion and MRE brain stiffness. Hetzer *et al*.^64^ provided evidence for this, reporting a significant correlation between perfusion and MRE brain stiffness. Consequently, the observed negative correlation between WMH and stiffness could be indicative of these perfusion disturbances influencing tissue microstructure. Areas with a higher burden of WMH might undergo altered perfusion, leading to tissue changes that manifest as reduced stiffness. It is worth mentioning that some studies have indicated that chronic cerebral hypoperfusion occurs even prior to the formation of WMH.^65-67^ Softer WMH brain regions may also simply represent inflammation. Studies have demonstrated that MRE can potentially uncover the mechanistic intricacies of neuroinflammation, making it a viable alternative to gadolinium-based contrast MRI.^68^ In the context of multiple sclerosis with WM lesions, recent studies involving both mouse models and human patients indicated that brain tissue softens as neuroinflammatory processes progress, revealing insights into acute inflammatory activity and the severity of the disease.^30,69^ Enhancement of perivascular spaces and detachment of astrocyte endfeet may be potential mechanisms that contribute to weaker neuronal–vascular network connections and, consequently, result in reduced tissue stiffness.^68,70^ Hypoperfusion induces the activation and degeneration of astrocytes, which ultimately leads to fibrosis of the ECM.^71^ Sweeney *et al*.^11^ described that cerebral hypoperfusion may disrupt the NVU and the integrity of the blood–brain barrier (BBB). This in turn contributes to demyelination, decreased synaptic plasticity and impaired brain function even before structural damage occurs.^72-74^ Eventually, the uncoupling of the NVU is accompanied by mitochondrial dysfunction and oxidative stress, neuronal death, changes in cerebral glucose metabolism and brain tissue atrophy.^75,76^ In this study, we also report the associations of brain stiffness with global cognitive performance, supporting the potential of mechanical biomarkers to help correlate with the cognitive outcomes during normal aging and in age-related disorders. Previous studies from Johnson *et al*. reported a significant negative correlation of memory performance with damping ratio of hippocampus and subfields in young adults.^36,77,78^ In a healthy aging population, they observed left hippocampal damping ratio is negatively associated with episodic memory performance.^79^ Our current study assesses the relation between brain stiffness and global cognition in an older cohort with a relatively wide range of stiffness. Our findings are in line with the recent studies by the same group^37^ where the relation between hippocampal subfield stiffness and cognitive function is reported in the healthy aging population.

Various cross-sectional and longitudinal studies have reported that global cognition change is strongly associated with brain grey matter volume change, with independent effects of global grey matter change and specific temporal lobe grey matter change.^80,81^ Depending on the processing methods used, partial volume effects can bias MRE-based stiffness measurements, especially in an older age population as shown in this study.^55^ This study utilized a neural network inversion designed to mitigate these partial volume effects by segmenting out displacement measurements from outside the brain prior to inversion and training the algorithm to estimate stiffness despite these missing measurements.^51^ Using the simulated data set previously published,^51^ we measured that global stiffness estimates were highly correlated with the ground truth but not significantly associated with total brain volume. On this basis, we expect that the findings reported here reflect primarily alterations in the brain parenchyma and not atrophy-related bias.

This study is a preliminary investigation to understand the association between vascular health, cognition and brain stiffness. The statistical analyses here are limited by the sample size. With additional data, a more sensitive region of interest for brain stiffness could be developed in an independent data set to improve sensitivity to the vascular measures. Although multiple markers of vascular health were explored, the impact of microinfarcts and microbleeds were not considered and will be the subject of future investigation. The coexistence of vascular and amyloid pathologies and the mechanisms linking these to cognitive decline needs further investigation.

## Conclusions

Strong associations of vascular markers and global cognitive function with stiffness establish the potential value of viscoelastic properties in understanding the vascular cognitive impairment. A statistically significant mediating role of stiffness on the indirect effects of WMH and CMC on cognition indicates the possible utilization of tissue mechanical alterations in probing microstructural damage and cognition decline. These results motivate a future longitudinal study exploring the mechanisms linking stiffness to cognitive decline in aging and dementia.

## Supplementary Material

fcae073_Supplementary_Data

## Data Availability

Clinical imaging data used in this study has not been made available to protect patient confidentiality in accordance with the Mayo Clinic institutional review board policies. In-house data processing and analysis scripts are available upon reasonable request after the completion of data sharing agreement.
