# Supplementary material for: Associations between vascular health, brain stiffness and global cognitive function
Source: Brain Commun. 2024 Feb 29;6(2):fcae073. doi: 10.1093/braincomms/fcae073 (PMC10950054; doi:10.1093/braincomms/fcae073)
Supplement: fcae073_Supplementary_Data [file fcae073_supplementary_data.pdf]

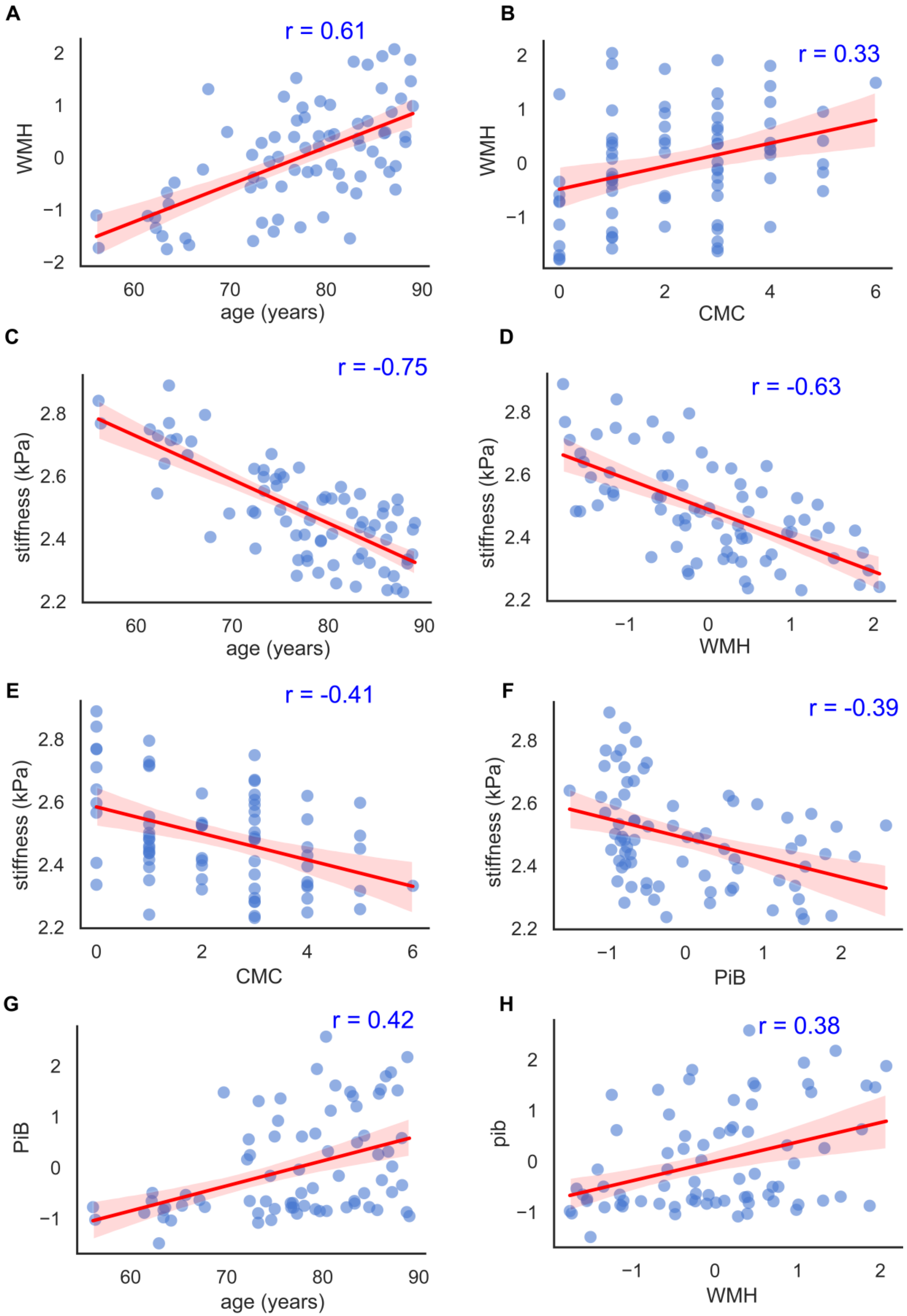

A
r = 0.61
WMH
age (years)
B
r = 0.33
WMH
CMC
C
r = -0.75
stiffness (kPa)
age (years)
D
r = -0.63
stiffness (kPa)
WMH
E
r = -0.41
stiffness (kPa)
CMC
F
r = -0.39
stiffness (kPa)
PiB
G
r = 0.42
PiB
age (years)
H
r = 0.38
pib
WMH

Supplementary Figure 1: Pearson correlations between the continuous variables. (A), (B) WMH versus age and CMC. **(C), (D), (E) and (F)** stiffness versus age, WMH, CMC and PiB**. (G), (H)** PiB versus age and WMH respectively. 'r' represents the Pearson correlation coefficient.

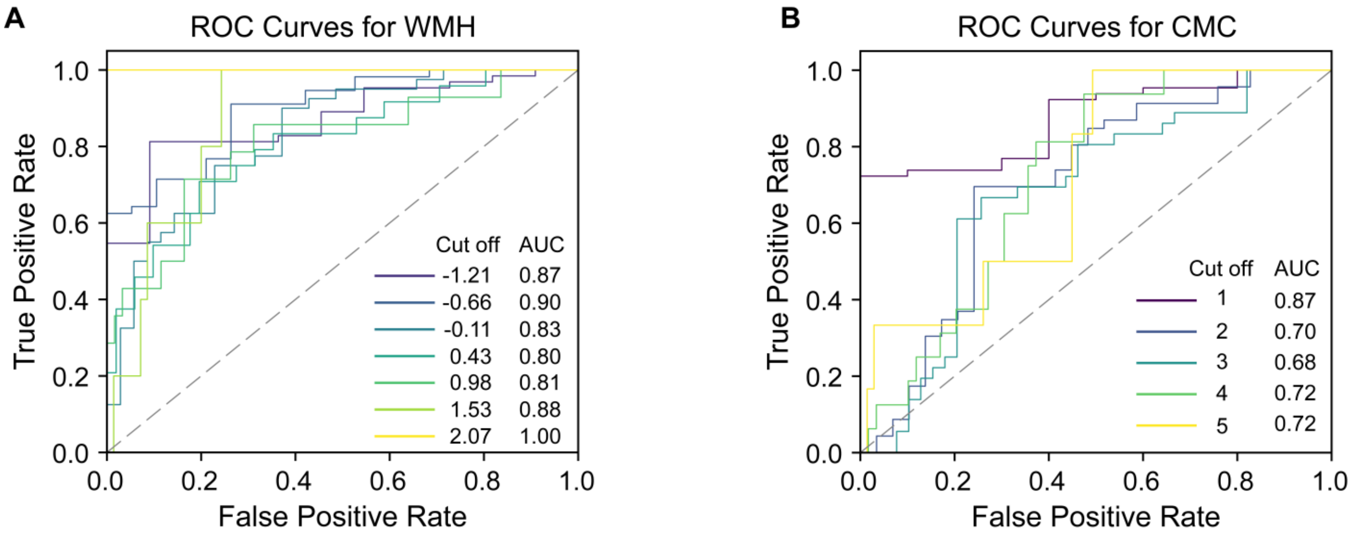

Supplementary Figure 2: Diagnostic accuracy of MRE stiffness for WMH and CMC. (A) Receiver Operator Characteristics for WMH illustrating the true positive rate (sensitivity) against the false positive rate (1-specificity) as the classification threshold for WMH varies. The diagonal line represents a random guess model, while our logistic regression model with age, sex, stiffness, CMC, and amyloid load as predictors lies above this line, indicating a better than random guessing performance. The Area Under Curve (AUC) of >80 suggests a good ability of the model to discriminate between the high and low WMH score groups. (**B**) Receiver Operator Characteristics for CMC at various thresholds. AUC >70 suggests an acceptable ability of the model to discriminate between low and high CMC using age, sex, brain stiffness, WMH and amyloid load as predictors.
